# Finding New Insights in Cardiac Resynchronization Therapy and the Pathophysiology behind Left Ventricular Dyssynchrony

**DOI:** 10.3390/jcm11226831

**Published:** 2022-11-18

**Authors:** Jürgen Duchenne

**Affiliations:** Department of Cardiovascular Sciences, KU Leuven—University of Leuven, 3000 Leuven, Belgium; jurgen.duchenne@kuleuven.be; Tel.: +32-16-342867; Fax: +32-16-344240

Over the past two decades, cardiac resynchronization therapy (CRT) became an established treatment option for patients with symptomatic heart failure. Current guideline criteria recommend implanting a CRT in patients with reduced left ventricular (LV) ejection fraction (LVEF ≤ 35%) and conduction delays (a QRS ≥ 130 ms on ECG)—preferably in those patients with a left bundle branch block (LBBB) morphology. Although the underlying problem is of an electrical nature—i.e., a myocardial conduction delay—the result in the left ventricle is of a mechanical nature: a dyssynchrony between the early activated septal wall and the late-activated lateral wall. Correcting this mechanical dyssynchrony by means of bi-ventricular pacing, in order to resynchronize the contraction pattern, is the main mode of action of CRT. Typically, CRT is considered successful when patients show an improvement in heart failure symptoms and/or reverse remodelling of their left ventricle (i.e., an increase in LV function or reduction in LV volume). Such patients are commonly labelled as ‘responder’.

One significant limitation of CRT is, however, the high rate of ‘non-response’. Recent data repeatedly indicate that about one out of three patients do not show an improvement after CRT [1]—even when selected according to guideline criteria and regardless of which outcome marker used. Another problem is the relative underutilization of CRT, due to the failure to recognize the need for CRT, as well as the lack of referral to CRT implantation.

*What Are Reasons for Non-Response?*

Two main problems can be identified: (I) the absence of a ‘treatable substrate’ for CRT, and (II) reasons that reduce the likelihood of response (either intrinsic to the heart or external). While improving the selection criteria for CRT has been a topic of controversy for several years—and continues to be so—reasons that reduce or even prevent response are still very much underexplored.

For a CRT device—which is aimed at resynchronizing the heart—the treatable substrate is the presence of dyssynchrony. Since about one out three patients currently implanted with a CRT do not show signs of mechanical dyssynchrony [2], it appears trivial that, in those patients, response is consequently low. Indeed, studies have repeatedly indicated that patients without the presence of mechanical dyssynchrony prior to CRT do not reverse remodel and have much lower chance of both short- and long-term survival after CRT. Nevertheless, guideline criteria do not recommend assessing the presence mechanical dyssynchrony prior to CRT implantation.

Whereas it is well established that the presence of myocardial scarring in the lateral wall of the left ventricle can severely hamper the efficacy of CRT—since scarred myocardium in the lateral wall would not transmit the electrical signal of the LV lead—the effect of scarring in other LV walls is understudied. Recent studies suggest that the presence of scarring in the septal wall also significantly impacts the ability to respond to CRT, since a scarred septum cannot recover contractile function during the remodelling process after CRT implantation [3,4]. While the assessment of myocardial scarring in heart failure patients has become clinical routine in many centres worldwide, it is not included (yet) as a selection criterion in the current guidelines for CRT implantation [5].

Some data suggest that a subgroup of patients exists with such severe LV dysfunction that they might have surpassed the optimal therapeutic window where CRT is most beneficial [6]. Indeed, since heart failure is a progressive disease and triggers compensatory mechanisms—ending in a viscous cycle—the inability to respond due to advanced cardiomyopathy does not appear surprising. Improved phenotyping may allow for a better response and survival prediction within the heart failure stages, but this remains to be explored.

Pacing-related issues are not to be underestimated as reason for poor response after CRT implantation. Whereas some problems can already be identified shortly after implantation—e.g., suboptimal atrio-ventricular timing, arrhythmias or suboptimal LV lead positioning—others require a longer follow-up, such as is the case of patients with loss of bi-ventricular pacing [7]. In such, even with optimal planning and selection, patients can still become non-responders.

Lastly, other intrinsic and external reasons for reduced response after CRT are still underexplored. Examples include the effect of (I) reduced right ventricular systolic function and/or increased pulmonary pressures; (II) diastolic dysfunction; (III) left atrial dyssynchrony; and (IV) anaemia.

*What Can We Do about the High Rate of Non-Response?*

Evidence is building that the selection criteria for CRT should be updated. Particularly assessing the presence of mechanical dyssynchrony in heart failure patients with conduction delays appears promising to select those patients in whom CRT will likely be successful. Evidence from large ongoing randomized trials—to be expected in the forthcoming years (e.g., the AMEND-CRT trial, ClinicalTrials.gov NCT04225520)—could further strengthen results from recent observational trials.

Another focus should be improving awareness that the presence of scarring in the left ventricle is an important determinant of non-response to CRT. The current gold standard for myocardial scar detection—cardiac magnetic resonance imaging (CMR) with late gadolinium enhancement (LGE)—is widely available, but currently still underused. Other potential alternatives to CMR—which is hampered by a relatively high cost and difficulty of use in patients with already implanted devices or severe renal dysfunction (both of which are common in CRT candidates)—should further be explored.

In addition to a potentially promising role for imaging in improving the selection of CRT candidates, new ECG-based markers also suggest that a more careful analysis of patients’ ECG could provide useful insights. A number of techniques—among which vectorcardiography—appear promising in selecting those patients likely to respond to CRT. Evidence from a large ongoing observational trial will provide more insight into their strength (e.g., the MARC-2 trial, ClinicalTrials.gov NCT04120909).

Perhaps even more important than new selection markers is further improving our understanding on the pathophysiology behind heart failure with conduction delays and mechanical dyssynchrony. With better insight, a better selection of patients—and thus a better responder/non-responder balance—can be expected. Particularly, a better serial follow-up of patients with conduction delays—even when they do not show signs of LV dysfunction—to monitor any potential remodelling as well as presence of mechanical dyssynchrony could provide more insight into the progressive deterioration caused by this disease.

Together with continued technological developments, hope exists to optimize the use of resynchronization therapy and to deliver this technique to all patients that could benefit from it.

In conclusion, this Special Issue in the *Journal of Clinical Medicine*—dedicated to “Finding New Insights in Cardiac Resynchronization Therapy and the Pathophysiology Behind Left Ventricular Dyssynchrony”—aims to pave the way towards the objectives provided above. We welcomed original research and review articles that: (1) shed new light on pathophysiology; (2) highlight and provide answers to unmet clinical needs; (3) provide insights that could expand or improve guideline indications; and (4) discuss future directions in research. Manuscripts with both a clinical and a translational focus (e.g., large animal research/applied computer models/machine learning) were considered.
